# Caveolin-1 Expression Increases upon Maturation in Dendritic Cells and Promotes Their Migration to Lymph Nodes Thereby Favoring the Induction of CD8^+^ T Cell Responses

**DOI:** 10.3389/fimmu.2017.01794

**Published:** 2017-12-13

**Authors:** Cesar Oyarce, Sebastián Cruz-Gomez, Felipe Galvez-Cancino, Pablo Vargas, Hélène D. Moreau, Natalia Diaz-Valdivia, Jorge Diaz, Flavio Andres Salazar-Onfray, Rodrigo Pacheco, Ana Maria Lennon-Dumenil, Andrew F. G. Quest, Alvaro Lladser

**Affiliations:** ^1^Laboratory of Gene Immunotherapy, Fundación Ciencia & Vida, Santiago, Chile; ^2^Laboratory of Cellular Communication, Advanced Center for Chronic Diseases (ACCDiS) and Center for Molecular Studies of the Cell (CEMC), Program in Cell and Molecular Biology, Faculty of Medicine, Biomedical Sciences Institute (ICBM), University of Chile, Santiago, Chile; ^3^Institut National de la Santé et de la Recherche Médicale Unité 144, Institut Curie/CNRS, Paris, France; ^4^Institut National de la Santé et de la Recherche Médicale Unité 932, Institut Curie/CNRS, Paris, France; ^5^Program in Immunology, Faculty of Medicine, Biomedical Sciences Institute (ICBM), University of Chile, Santiago, Chile; ^6^Laboratory of Neuroimmunology, Fundación Ciencia & Vida, Santiago, Chile; ^7^Departamento de Ciencias Biológicas, Facultad de Ciencias Biológicas, Universidad Andres Bello, Santiago, Chile

**Keywords:** caveolin-1, dendritic cells, migration, chemotaxis, CD8^+^ T cell activation, antitumor immune response

## Abstract

Dendritic cell (DC) trafficking from peripheral tissues to lymph nodes (LNs) is a key step required to initiate T cell responses against pathogens as well as tumors. In this context, cellular membrane protrusions and the actin cytoskeleton are essential to guide DC migration towards chemotactic signals. Caveolin-1 (CAV1) is a scaffolding protein that modulates signaling pathways leading to remodeling of the actin cytoskeleton and enhanced migration of cancer cells. However, whether CAV1 is relevant for DC function and specifically for DC migration to LNs is unknown. Here, we show that CAV1 expression is upregulated in DCs upon LPS- and TNF-α-induced maturation. CAV1 deficiency did not affect differentiation, maturation, or the ability of DCs to activate CD8^+^ T cells *in vitro*. However, CAV1-deficient (CAV1^−/−^) DCs displayed reduced *in vivo* trafficking to draining LNs in control and inflammatory conditions. *In vitro*, CAV1^−/−^ DCs showed reduced directional migration in CCL21 gradients in transwell assays without affecting migration velocity in confined microchannels or three-dimensional collagen matrices. In addition, CAV1^−/−^ DCs displayed reduced activation of the small GTPase Rac1, a regulator of actin cytoskeletal remodeling, and lower numbers of F-actin-forming protrusions. Furthermore, mice adoptively transferred with peptide-pulsed CAV1^−/−^ DCs showed reduced CD8^+^ T cell responses and antitumor protection. Our results suggest that CAV1 promotes the activation of Rac1 and the formation of membrane protrusions that favor DC chemotactic trafficking toward LNs where they can initiate cytotoxic T cell responses.

## Introduction

Dendritic cells (DCs) are professional antigen-presenting cells specialized in initiating adaptive T cell responses. DCs circulate and patrol peripheral tissues, taking up protein antigens and processing them into small peptides that are presented at the cell surface by major histocompatibility complex (MHC) molecules. After recognition of pathogen- or danger-associated signals, DCs maturate and migrate *via* lymphatic vessels to secondary lymphoid organs [i.e., lymph nodes (LNs)] where they activate antigen-specific naïve T cells (1). DC maturation induces upregulation of several proteins, including co-stimulatory molecules and cytokines (2) and also increases DC trafficking toward secondary lymphoid organs by increasing polarized migration and upregulating chemokine receptors, such as CCR7 (3, 4). Increased CCR7 expression allows DCs to detect increasing concentrations of CCL19/CCL21 (5, 6), which promotes haptotactic DC migration to the lymph vessels and entering into T cell rich areas of LNs (*paracortex*) (7, 8). Trafficking of DCs from peripheral tissues to LNs involves several sequential steps: (1) mobilization, (2) detachment, (3) interstitial migration, (4) entry into the afferent lymphatics, and (5) transit *via* the lymph (9).

To migrate through epithelial barriers, DCs extend F-actin membrane protrusions at the cell front to associate *via* integrins with extracellular substrates. These points of contact are coupled to the cytoskeleton to transduce the internal force that is generated when myosin II contracts the actin network, allowing retrograde traction forces on the integrins to move the cell. Then to migrate through three-dimensional matrices, DCs use adhesion-independent amoeboid migration, which is driven by protrusive flowing of the actin network at the leading edge of the cell. Myosin II-dependent contraction of the trailing edge is required when DCs need to pass through narrow gaps. On their way to LNs, DCs also need to transmigrate into lymph vessels (3) and proteins expressed in the lymph vessels promote actomyosin-mediated cellular contraction in DCs (10, 11), thereby enhancing cell migration across the lymphatic endothelium (12). Once DCs reach the lumen of lymph vessels, chemokine signals like CCL21 gradients (13) and mechanical forces like hydrostatic pressure or friction (14) guide the “squeezing and flowing” of the actin cytoskeleton that defines amoeboid DC migration (13). Finally, DCs enter the LN and transmigrate to the *paracortex* (T cell rich area) (15), where they activate T cells. As indicated above, regulation of actin cytoskeleton remodeling is important in every step of DC trafficking (14). Indeed, it has been suggested that actin flow may determine cell speed and persistency (16), highlighting the importance of actin cytoskeleton dynamics during DC trafficking. Such fine-tuned control is achieved mostly by the small GTPases Rho (17), Cdc42 (18), and Rac1 (19). However, despite recent progress in this field, our understanding of these events in DCs is limited, and additional pathways or molecules that promote DC trafficking remain to be defined.

Caveolin-1 (CAV1) is a membrane-bound scaffolding protein implicated in caveolae formation (20) that interacts with and controls the activity of a large number of proteins involved in signaling pathways relevant to growth, survival and proliferation in different cell types (21–24). Accumulating evidence supports a role for CAV1 in cell migration. Indeed, it was shown that directional persistency and chemotaxis are reduced in CAV1-deficient fibroblasts (25). In cancer cells, CAV1 expression promotes cell migration and invasion *in vitro* (26, 27) and metastasis *in vivo* (28, 29). The molecular mechanisms that operate downstream of CAV1 in these models, involve an increase in Rac1 activity *via* activation of the recently identified CAV1/p85α/Rab5/Tiam1/Rac1 signaling axis (27). It was largely assumed that caveolin proteins were not expressed in leukocytes. However, emerging evidence indicates that they can be found in myeloid and, in some particular cases, lymphoid cells (30, 31). A few reports have shown CAV1 expression in DCs, but its role remains unclear. Some reports suggest that CAV1 is involved in caveolae-dependent endocytosis (32, 33). Another study suggests that CAV1 recruits and suppresses iNOS, thereby decreasing NO production and suppressing DC function during HSV-1 infection (34). Also, CAV1 has been shown to promote HIV-1 capture and lysosomal degradation by Langerhans cells (LCs), restricting viral integration and subsequent spreading (35). Interestingly, stimulation of human LCs with TNF-α increased CAV1 transcript levels (36), suggesting that CAV1 expression may be upregulated upon maturation. Taken together, these observations suggest that CAV1 might be relevant for DC function by modulating their migratory capacity.

In this study, we describe for the first time that CAV1 expression is upregulated upon DC maturation. Using CAV1-deficient (CAV1^−/−^) mice, we show that CAV1^−/−^ DCs displayed reduced *in vivo* trafficking to draining LNs in steady state and inflammatory conditions. CAV1^−/−^ DCs showed reduced migration toward CCL21 gradients in transwell assays, decreased Rac1 activity and lower numbers of F-actin-forming protrusions. Furthermore, peptide-pulsed CAV1^−/−^ DCs elicited reduced CD8^+^ T cell responses *in vivo* and poorer antitumor protection. Overall, our results suggest that CAV1 promotes migration of DCs to LNs, likely through Rac1-dependent actin cytoskeleton remodeling, to elicit effective T cell responses.

## Results

### CAV1 Expression is Upregulated upon DC Maturation

**Figure 1:**
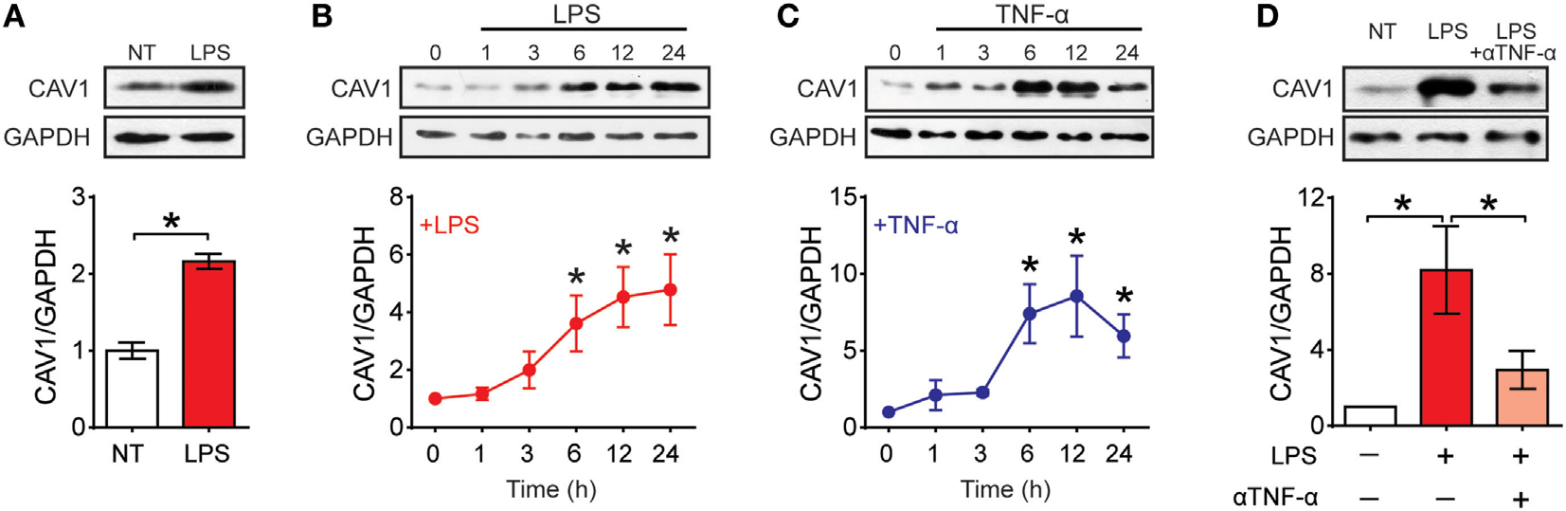
Caveolin-1 (CAV1) is expressed in dendritic cells (DCs) and upregulated upon maturation. CAV1 expression in DCs was assessed by Western blotting. GAPDH and actin were used as loading controls. CAV1 protein expression was quantified by densitometry analysis and standardized to loading control. Values normalized to untreated controls (NT) are shown. **(A)** Spleen DCs purified from wild-type mice were treated with LPS (100 ng/ml) for 6 h. **(B,C)** Bone marrow-derived DCs (BM-DCs) were stimulated with LPS (100 ng/ml) or TNF-α (20 ng/ml) for 0, 1, 3, 6, 12, or 24 h. **(D)** BM-DCs were treated with LPS (100 ng/ml) alone or in combination with a TNF-α blocking antibody (5 µg/ml) for 24 h. Values are presented as the mean ± SEM. *p < 0.05; *n* = 3 independent experiments.

To determine what happens to CAV1 expression upon maturation, we first evaluated by Western blot analysis CAV1 expression in purified spleen DCs (Sp-DCs) and bone marrow-derived DCs (BM-DCs) following stimulation with LPS and TNF-α (Figure 1; Figures S1A,B in Supplementary Material). CAV1 expression was increased in Sp-DCs after 6 h of LPS stimulation (Figures 1A; Figure S1A in Supplementary Material). Both LPS and TNF-α induced a time-dependent increase in CAV1 expression following 6–24 h of stimulation in BM-DCs (Figures 1B,C; Figure S1B in Supplementary Material). Since TNF-α secretion is induced by LPS (Figure S1C in Supplementary Material), we next evaluated whether autocrine TNF-α was involved in LPS-induced CAV1 upregulation. To this end, TNF-α was blocked using a neutralizing antibody present for 6- or 24 h during LPS stimulation. As shown in Figure 1D, the TNF-α blocking antibody reduced by over 50% CAV1 upregulation after 24 h stimulation. However, blocking TNF-α did not change LPS-induced upregulation of CAV1 at an earlier time point (6 h) following LPS treatment (Figure S1D in Supplementary Material), indicating that LPS-induced upregulation of CAV1 initially does not require TNF-α, but that secreted TNF-α then plays a predominant role at later time points.

To assess the role of CAV1 in DC function, we used a CAV1 null (CAV1^−/−^) mouse model (37). To validate the model, we first determined the frequency of DCs present in spleen, LNs and skin (as an example of peripheral tissue). We found that DC frequency was the same in both CAV1^−/−^ and wild-type (WT) mice for all the tested tissues (Figures S2A–C in Supplementary Material). Moreover, no differences were observed in terms of viability, differentiation, expression of lineage markers, and co-stimulatory molecules, as well as cytokine secretion when comparing BM-DCs generated from CAV1^−/−^ with DCs from WT mice (Figure S2D in Supplementary Material). We also evaluated co-stimulatory molecule expression and neither CD14, CD40, CD86, CD38, PD-L1 nor MHC-I expression changed when comparing WT and CAV1^−/−^ DCs, irrespective of their maturation status. The only difference detected was a minor reduction in CD80 and CCR7 expression in immature CAV1^−/−^ DCs when compared with WT DCs. This difference was not evident when analyzing mature DCs though. Furthermore, cytokine secretion (IL-6, IL-12, and TNF-α) was the same for control or LPS-maturated WT or CAV1^−/−^ DCs. Given that a key feature of DCs is to activate T cells, we evaluated whether CAV1 participates in DC-mediated CD8^+^ T cell activation. In agreement with our previous data, both WT and CAV1^−/−^ DCs pulsed with different concentrations of the OVA_257–264_ peptide (SIINFEKL) were equally efficient at inducing the proliferation of CFSE-stained OVA_257–264_-specific OT-I CD8^+^ T cells (Figure S3A in Supplementary Material). These results suggest that CAV1 deficiency in DCs does not affect either differentiation, maturation or the ability to activate CD8^+^ T cells *in vitro*.

### CAV1 Promotes DC Trafficking to LNs

**Figure 2:**
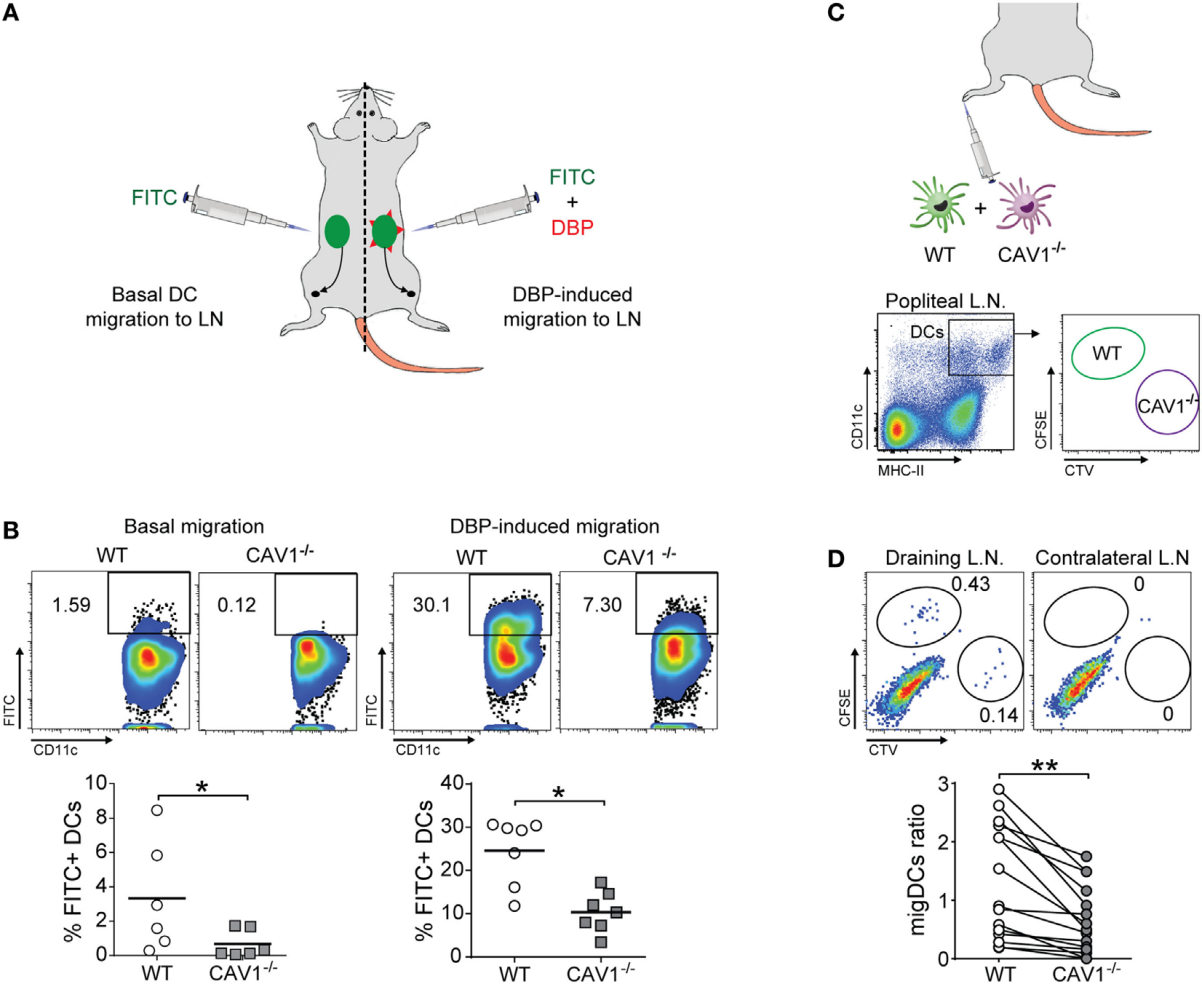
Caveolin-1 (CAV1) favors dendritic cell (DC) trafficking to lymph nodes (LNs) *in vivo* **(A,B)**. Back skin of wild-type (WT) and CAV1^−/−^ mice were treated with FITC (left flank) or FITC + dibutyl phthalate (DBP) (right flank), as summarized in **(A)**. **(B)** After 24 h, the arrival of skin-derived FITC^+^ DCs to inguinal LNs was evaluated. Representative density plots (top panels) and quantification of FITC^+^ DCs (bottom panels) under basal or DBP-induced conditions are shown. Each dot represents one animal, and the bar is the mean (*p < 0.05, *n* = 7). **(C,D)** WT and CAV1^−/−^ bone marrow-derived DCs (BM-DCs) were stained with CFSE and Cell Trace Violet (CTV), respectively. Then, WT recipient mice were subcutaneously injected in the right footpad with 5 × 10^5^ cells (1:1 ratio, WT to CAV1^−/−^) or with PBS as a control (left footpad). The arrival of CFSE (WT) or CTV (CAV1^−/−^) BM-DCs to the draining (right) and contralateral (left) popliteal LNs was evaluated 24 h later. **(C)** Scheme of footpad injection and gating analysis to analyze transferred BM-DCs. **(D)** Representative dot plots of DCs in the draining popliteal LNs are shown. Gates showing injected WT and CAV1^−/−^ DCs are displayed. The migration index of WT or CAV1^−/−^ DCs was calculated as {[% CTV stained DC in popliteal lymph nodes (PLN)]/(% CTV stained DC in input)}/[(% CFSE WT DC in PLN)/(% CFSE WT DC in input)]. Data are presented as dot plots with connecting lines per paired samples. **p < 0.01, *n* = 16 mice, from three independent experiments.

To identify a possible role for CAV1 in DC function *in vivo*, we tested whether CAV1 was involved in the trafficking of DCs from the periphery to LNs by performing the FITC painting assay. To this end, mice were inoculated with a FITC-containing solution at two different sites (left and right) of the lower back (38). To further induce inflammation and enhance skin DC migration, one site was additionally treated with dibutyl phthalate (DBP), which is a well-known skin irritant that promotes DC migration (39). As summarized in the scheme (Figure 2A), the accumulation of FITC-positive migratory DCs (FITC^+^CD11c^+^MHC-II^high^) in the inguinal LN was evaluated by flow cytometry after 20 h. Viable cells were gated for co-expression of CD11c and MHC-II to identify DCs (gating strategy shown in Figure S2D in Supplementary Material). As shown, the absence of CAV1 drastically impaired DC trafficking to the inguinal LNs in both control and inflammatory conditions (Figure 2B). Indeed, 1.6% of WT DCs reached LNs as compared with only 0.1% for CAV1^−/−^ DCs, indicating that the lack of CAV1 in DCs impaired almost completely migration in the control condition (Figure 2B, left panels). DBP-induced migration of WT DCs increased up to 25% as compared with only 10% for CAV1^−/−^ DCs (Figure 2B, right panels). These results suggest that CAV1 expression is fundamental for DC trafficking from skin to the draining LNs.

As DC prevalence was similar in the skin of WT and CAV1^−/−^ mice (Figure S2C in Supplementary Material), impaired DC trafficking to the LNs in CAV1^−/−^ mice could not be attributed to reduced numbers of skin DCs. However, because these experiments were performed in mice that lacked the expression of CAV1 in all cell types, the effects observed for DCs may be attributed to changes in the environment rather than the DCs themselves. Thus, we performed an experiment where CAV1 deficiency was restricted just to DCs. WT and CAV1^−/−^ BM-DCs were labeled with carboxyfluorescein succinimidyl (CFSE) or *Cell Trace Violet* (CTV), respectively, mixed at a 1:1 ratio and then injected into the footpad of recipient WT mice. After 24 h, both draining and contralateral (control) popliteal LNs were obtained and processed to analyze the presence of transferred WT (CFSE-positive) and CAV1^−/−^ (CTV-positive) DCs by flow cytometry, as depicted in the scheme (Figure 2C, gating strategy similar to Figure S2B in Supplementary Material). As shown (Figure 2D), the frequency of CAV1^−/−^ DCs in the draining popliteal LNs was reduced by nearly 50% compared with WT DCs. Neither WT nor CAV1^−/−^ DCs were detected in the contralateral LNs. Taken together, these findings indicate that CAV1 intrinsically modulates the migratory behavior of DCs by promoting their trafficking to LNs, which represents a fundamental step to initiate adaptive immune responses.

### CAV1 Promotes DC Transmigration

**Figure 3:**
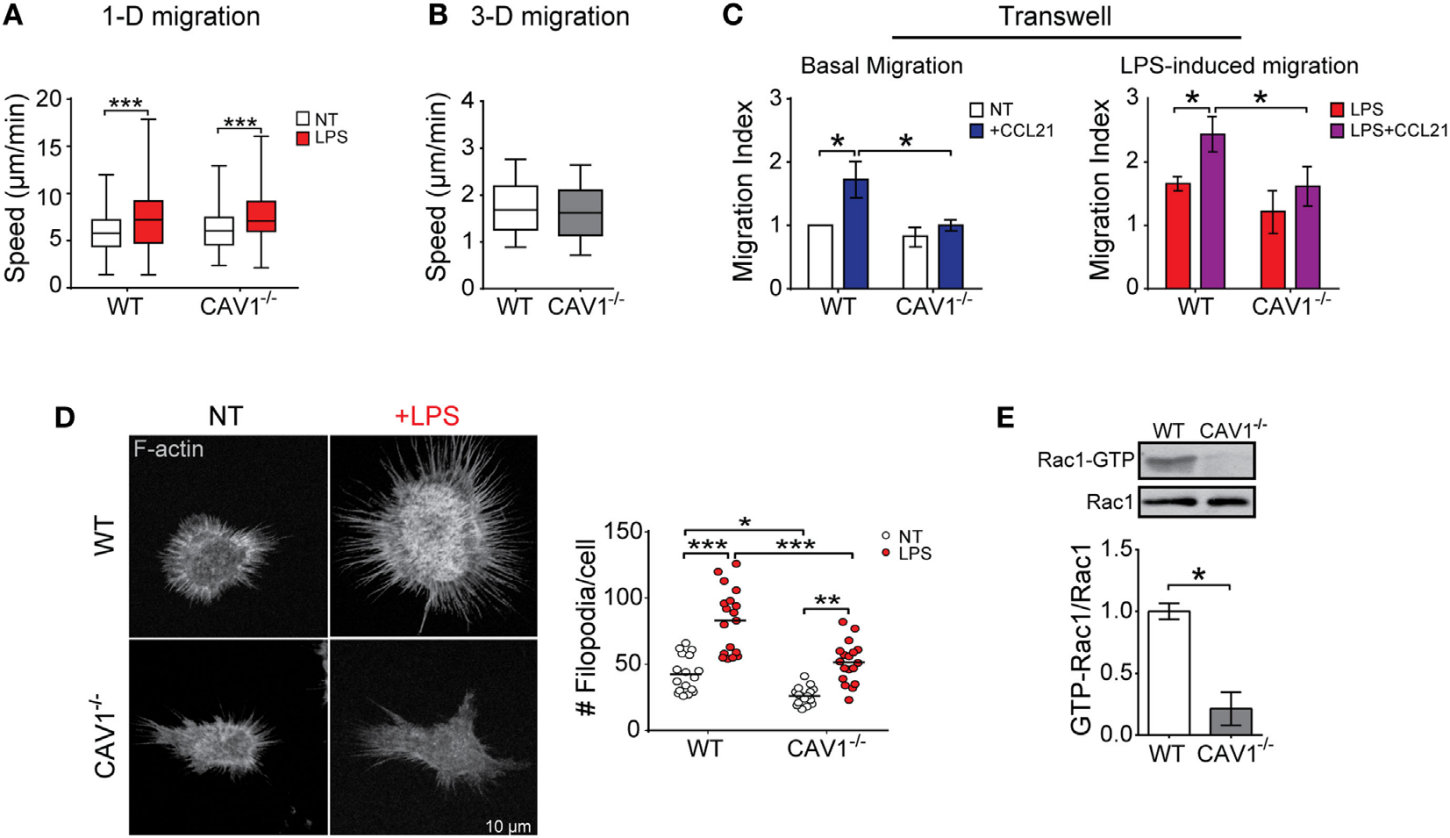
Caveolin-1 (CAV1) promotes dendritic cell (DC) transmigration, actin membrane protrusions, and Rac1 activation. Migration of wild-type (WT) or CAV1^−/−^ bone marrow-derived DCs (BM-DCs) in different *in vitro* assays. **(A)** BM-DCs treated or not with LPS (1 µg/ml, for 30 min) were individually tracked in confined microchannels (4 µm). After 5–6 h, cell images at different positions along the channels were recorded during 10–12 h (one photo each 2 min) using an automated microscope. The reconstructed movie is analyzed, and the average speed of every cell obtained. In the box plots, the bars include 90% of the data points, the center corresponds to the median, and the box contains 75% of the data points. Data from two different experiments, *n* = WT: 221, WT + LPS: 153, CAV1^−/−^: 120, CAV1^−/−^ + LPS: 236 cells (***p < 0.001). **(B)** Chemotactic migration of LPS-treated WT or CAV1^−/−^ BM-DCs embedded in a 1% bovine collagen gel containing a CCL21 gradient. Mean velocity of LPS-DCs depicted as a function of the distance to the CCL21 source. In the box plots, the bars include 90% of the data points, the center corresponds to the median, and the box contains 75% of the data points. *n* = WT: 1,090, CAV1^−/−^: 1,169 tracks, from two independent experiments. **(C)** DC transwell migration. The bottom side of transwell membranes was coated with fibronectin to avoid losing migratory cell. In the bottom chamber, RPMI medium containing 0.5% fetal bovine serum with or without CCL21 (20 ng/ml) was added. Then, 2 × 10^5^ of WT or CAV1^−/−^ BM-DCs were seeded in the upper chamber, and migration was evaluated by counting cells on the bottom surface of the membrane. Left panel, immature BM-DC migration after 1 h. Right panel, LPS-matured BM-DC migration after 30 min. The migration index (relative to spontaneous WT DC migration) is shown. Bars are mean ± SEM (*p < 0.05, *n* = 3). **(D)** In the left panel, representative confocal microscopy images showing F-actin (phalloidin staining) in WT or CAV1^−/−^ BM-DCs treated or not with LPS (100 ng/ml, 24 h). To the right, the quantification of protrusions per cell is shown. Each dot corresponds to one cell, and the black bar represents the mean (*p < 0.05, **p < 0.01, and ***p < 0.001, *n* = 18 cells, two experiments). **(E)** GTP-Rac1 levels were determined in WT and CAV1^−/−^ DCs using pull-down assay followed by Western blot. Representative blots showing the active GTP-bound fraction and total Rac1. The ratio between active and total Rac1 is shown in the plot (densitometry analysis). Data are the mean ± SD (*p < 0.05, *n* = 3).

To shed light on the mechanisms by which CAV1 favors DC trafficking to LNs, we employed *in vitro* assays that represent the different types of migration that enable DCs to reach secondary lymphoid organs (14): confined unidirectional migration (microchannels), three-dimensional (collagen matrix), and two-dimensional migration (transwell). While microchannels mimic the confined spaces typically present in peripheral tissues (40), collagen matrix migration resembles amoeboid interstitial migration and transwell assays emulate entry to lymphatic vessels and transmigration across lymphatic endothelium. We first evaluated whether CAV1 modulates DC trafficking using fabricated microchannels, as well as in collagen matrix using CCL21 as a chemoattractant. Migration velocities in both assays were similar for WT and CAV1^−/−^ DCs (Figures 3A,B), indicating that intrinsic DC motility is independent of CAV1. To enter lymphatic vessels, DCs must pass through narrow openings -loose flaps of about 2–3 µm in diameter—present at the beginning of initial lymphatic capillaries (41), in a process that requires direct contact with endothelial cells and matrix (42). Then, DCs transmigrate across lymphatic endothelium to reach the LNs (43). Hence, to determine if CAV1 was involved in facilitating these processes, a transwell migration assay was performed. As shown (Figure 3C, left panel), basal DC transmigration induced by exposure to CCL21 was severely reduced in CAV1^−/−^ DCs. Moreover, LPS-induced transmigration was also reduced in CAV1^−/−^ DCs (Figure 3C, right panel). Taken together, these observations suggested that CAV1 promotes DC trafficking to LNs by increasing transmigration.

It has been suggested that during DC transmigration, the cells actively push open the junction to enter the lymphatic capillary (44). As actin cytoskeleton protrusions could be involved in the junction opening and transmigration across lymphatic endothelium, we evaluated the role of CAV1 in the formation of membrane protrusions. As shown (Figure 3D, left panel), actin microfilament staining using phalloidin revealed a reduced number of actin-based membrane protrusions for immature CAV1^−/−^ DCs as compared with WT cells. LPS increased significantly membrane protrusions in WT DCs; however, in CAV1^−/−^ DCs almost 40% fewer projections were detected, suggesting that CAV1 promotes remodeling of the actin cytoskeleton in DCs. Previous reports have implicated the small GTPase Rac1 in actin cytoskeleton remodeling and formation of membrane protrusions in DCs (45), indicating that Rac1 inhibition decreased DC arrival to LNs (19). Therefore, Rac1 activity was determined in WT and CAV1^−/−^ DCs by a pull-down assay that uses a GST-PAK1 fusion protein to immunoprecipitate GTP-bound active Rac1. Then, Rac1 levels present in the pull-down fraction (Rac1-GTP), and total DC lysates were analyzed by Western blotting. As shown (Figure 3E), Rac1 activation was severely reduced in CAV1^−/−^ compared with WT DCs, thereby implicating CAV1 in Rac1 activation in DCs. Taken together, our results suggest that CAV1 promotes DC migration to the LNs by increasing DC transmigration, likely through Rac1-mediated actin cytoskeleton remodeling.

### CAV1 Enables DCs to Generate Tumor-Protective CD8^+^ T Cell Responses

**Figure 4:**
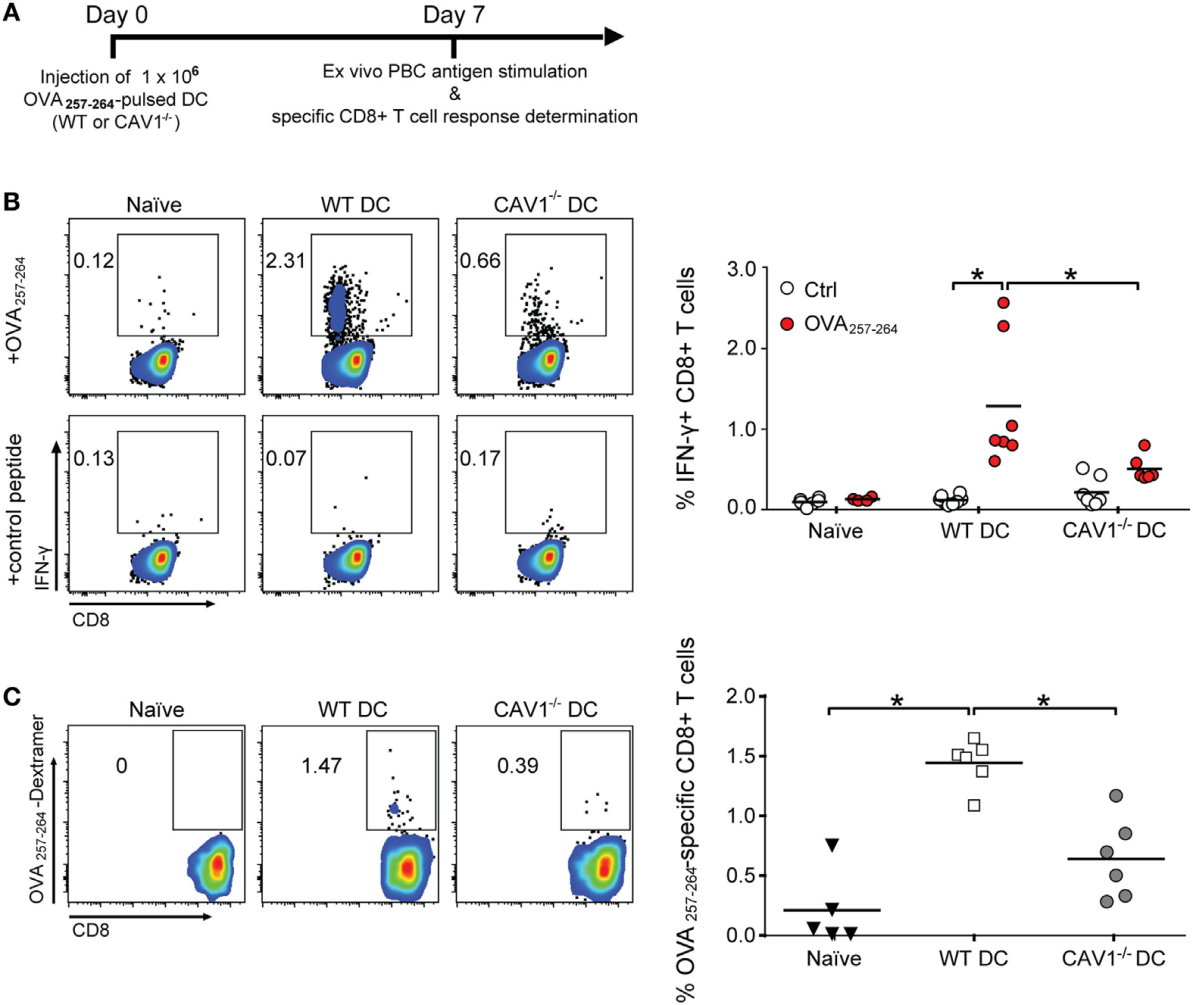
Caveolin-1 (CAV1) promotes the ability of dendritic cells (DCs) to induce antigen-specific CD8^+^ T cell responses. **(A)** Scheme of the experimental procedure. At day 0, 1 × 10^6^ OVA_257–265_-pulsed wild-type (WT) or CAV1^−/−^ bone marrow-derived DCs (BM-DCs) were transferred to recipient WT mice. After 7 days, blood samples were taken, and CD8^+^ T cell responses were analyzed. **(B)** Cells were stimulated with control (trp2_180–188_) or OVA_257–265_ peptides for 2 h, and then brefeldin A-containing solution (Golgi plug) was added for another 6 h (8 h total stimulation). Afterward, cells were stained and analyzed by flow cytometry. Representative dot plots of IFN-γ expression on gated CD3^++^CD8^+^ T cell population and the percentage of IFN-γ-producing CD8^+^ T cells are shown. Bars are the mean ± SEM (*p < 0.05, *n* = 7 mice, from two independent experiments). **(C)** Freshly isolated cells were stained with H-2 Kb/SIINFEKL (OVA_257–265_ peptide) dextramer to determine antigen-specific CD8^+^ T cells. Representative density plots and quantification of frequency of dextramer-positive from totalCD3^+^CD8^+^ T cell population are shown. Data are the mean ± SEM (*p < 0.05, *n* = 5–6 mice from two independent experiments).

To assess the potential consequences of CAV1 in promoting DC trafficking to the LNs, we evaluated the ability of DCs to initiate antigen-specific CD8^+^ T cell responses *in vivo* (46). Therefore, WT recipient mice were transferred with WT and CAV1^−/−^ DCs pulsed with OVA_257–264_ peptide to elicit CD8^+^ T cell responses independently of antigen uptake, processing, and presentation. Seven days later, OVA_257–264_-specific CD8^+^ T cell responses were determined in peripheral blood by *ex vivo* peptide stimulation followed by intracellular IFN-γ staining and flow cytometry analysis (see scheme in Figure 4A). As shown (Figure 4B), higher frequencies of IFN-γ-producing CD8^+^ T cells in response to OVA_257–264_ *ex vivo* stimulation were detected for WT DC-immunized mice compared with the CAV1^−/−^ DC-immunized group. To confirm that CAV1^−/−^ DCs elicited reduced CD8^+^ T cell responses, and not CD8^+^ T cells with an impaired ability to produce IFN-γ, the total frequencies of OVA_257–264_-specific CD8^+^ T cells were determined by staining with a H-2Kb/OVA_257–264_ multimer, which labels OVA_257–264_-specific CD8^+^ T cells regardless of the ability to respond to *ex vivo* peptide stimulation. As anticipated, reduced frequencies of H-2Kb/OVA_257–264_-specific CD8^+^ T cells were detected in mice immunized with CAV1^−/−^ DCs as compared with WT DCs (Figure 4C). Given our results showing that WT and CAV1^−/−^ DCs activate CD8^+^ T cells equally well when they interact *in vitro* (Figure S3A in Supplementary Material), these results suggest that the impaired arrival of CAV1^−/−^ DCs to lymphoid organs leads to reduced CD8^+^ T cell responses.

**Figure 5:**
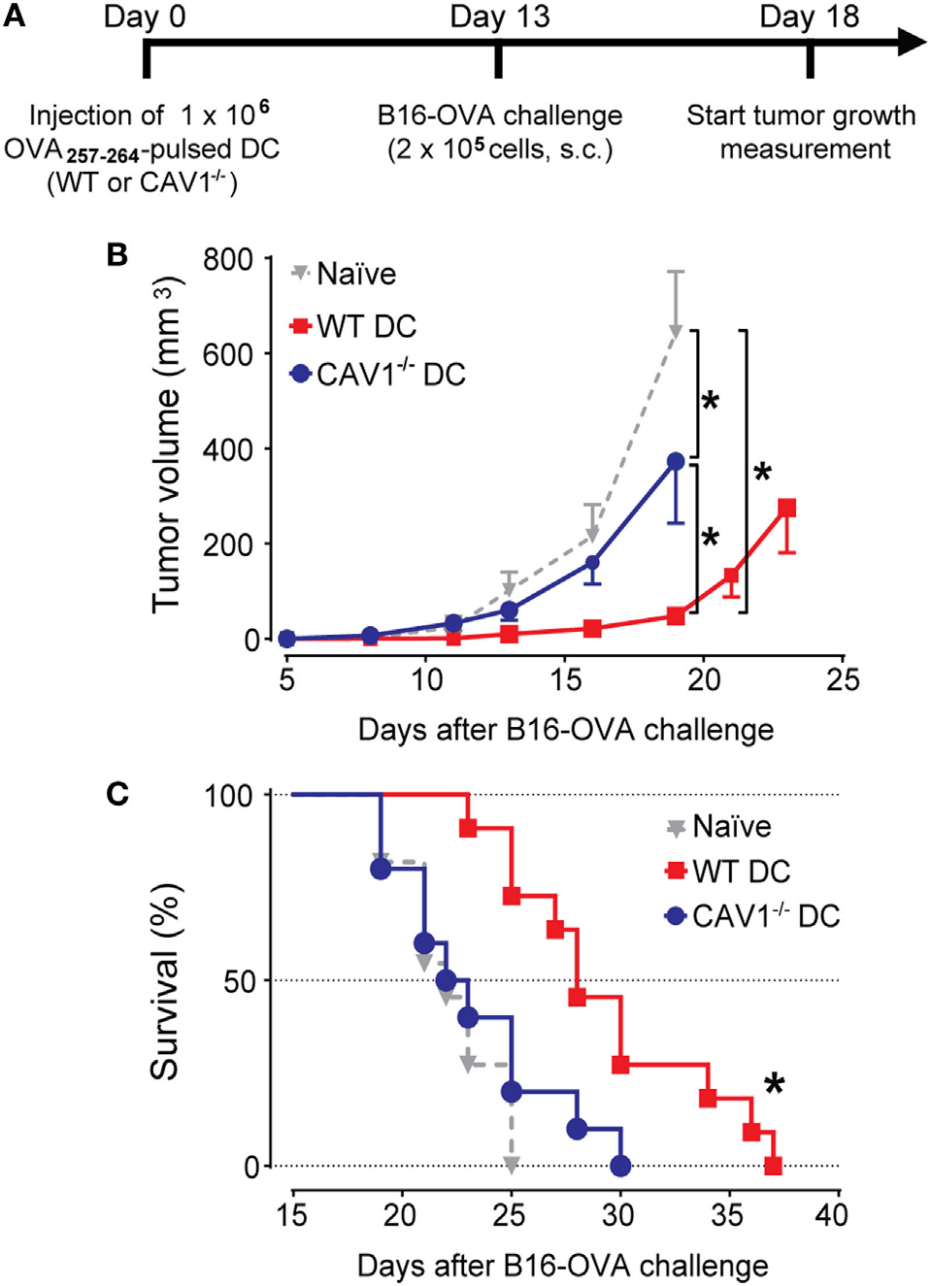
Caveolin-1 (CAV1) promotes dendritic cell (DC)-mediated antitumor protection. **(A)** Scheme showing experimental procedure. Initially, 1 × 10^6^ OVA_257–265_-pulsed wild-type (WT) or CAV1^−/−^ BM-DCs were transferred to WT recipient mice. The control group was injected with PBS (vehicle). After 13 days, mice were challenged s.c. with 2 × 10^5^ B16F10-OVA (B16-OVA) cells injected into the right flank. Tumor growth evaluation started 5 days after challenge and was carried out until all the animals were dead. The animals were sacrificed when the length, width, or height surpassed 15 mm. Groups were defined as: control mice group (gray triangles), OVA_257–265_-loaded WT (WT DCs, red squares), and OVA_257–265_-loaded CAV1^−/−^ DCs (CAV1^−/−^ DCs, blue circles). **(B)** Average tumor growth curves are shown (*p < 0.05, *t*-test, *n* = 8–10 mice). **(C)** Survival curves. The mean survival times were as follows: Ctrl group, 22 days; WT DC group, 28 days; CAV1^−/−^ DC group, 22,5 days (*p < 0.05, *n* = 8–10 mice, three different experiments).

To validate these results in a functional model that relies on the cytotoxic activity of specific CD8^+^ T cells *in vivo*, we performed tumor challenge experiments. To this end, recipient WT mice transferred with OVA_257–264_-loaded WT or CAV1^−/−^ DCs were challenged with ovalbumin-expressing B16 (B16-OVA) melanoma cells, and tumor growth was monitored every 2–3 days (Figure 5A). Significant suppression of tumor growth (Figure 5B) and extended survival (Figure 5C) were observed in mice receiving WT DCs as compared with control-treated (Ctrl) mice. By contrast, tumor suppression and survival were severely compromised in mice that received CAV1^−/−^ DCs. These results indicate that CAV1 promotes the ability of adoptively transferred DCs to initiate tumor-protective CD8^+^ T cell responses. Overall, our data identify a novel role for CAV1 in DC function by promoting DC trafficking to the LNs to efficiently initiate protective cytotoxic CD8^+^ T cell responses.

## Discussion

In this study, we show that CAV1 expression is upregulated in DCs upon maturation and plays a pivotal role in promoting the trafficking of DCs to LNs, a crucial step to initiate protective adaptive T cell responses. Furthermore, our data support the notion that CAV1 increases DC trafficking by enhancing transmigration, likely through Rac1-dependent remodeling of actin cytoskeleton. These findings unveil a novel function for CAV1 in DCs with a relevant effect in CTL-mediated responses.

Although it was largely assumed that CAV1 was not expressed in leukocytes, some studies indicated that it may be expressed in the myeloid compartment (30). Previous studies had described the presence of CAV1 in macrophages (47) and DCs (32–34) where the protein played contradictory roles in virus–host interaction. In DCs, CAV1 has been shown to prevent HIV virus infection (35), as well as to dampen host antiviral responses mediated by nitric oxide production (34). However, none of these studies evaluated changes in CAV1 expression upon maturation, its role in migration or initiation of adaptive immune responses. Here, we show that CAV1 is expressed in DCs at steady state, and progressively upregulated upon maturation induced by LPS or TNF-α. Moreover, LPS-induced CAV1 upregulation is reinforced at later time points in an autocrine manner by TNF-α (Figure 1; Figure S1 in Supplementary Material). Thus, it seems that TLR4 and TNFR signaling are relevant for CAV1 expression. Given that NF-κB is a master regulator of TLR- and TNF-α-dependent DC maturation (48), as well as CAV1 expression in other cell types (49), our data suggest that NF-κB may participate in the control of CAV1 expression in DCs. First, LPS is known to induce NF-κB activation *via* TLR4 signaling (50), resulting in TNF-α production. Second, TNF-α activates the TNF receptor that can also lead to NF-κB activation (51, 52). Interestingly, TNF-α was described to increase CAV1 transcript levels in human LC (36), a well-known migratory DC subset (53). Taken together, the previously published results and our findings suggest that maturation is linked to CAV1 upregulation in DCs, probably *via* NF-κB. Therefore, CAV1 upregulation is likely to represent a rather common event in DC biology.

In addition to its well-established role in endocytosis, an emerging role in cell migration has been ascribed to CAV1. However, such a role in immune cells, and particularly in DCs, had not been described yet. Here, we demonstrate that CAV1 promotes migration to draining LNs using two complementary *in vivo* models. Both endogenous CAV1^−/−^ DCs in knockout mice, as well as CAV1^−/−^ DCs transferred into WT mice, displayed impaired trafficking to LNs compared with their WT DC counterpart. These *in vivo* assays show that, even under inflammatory conditions, a major proportion of DCs remain in the tissue and only a few of them are able to find their way through lymph vessels to the draining LNs, inefficiently reaching more distant tissues (6). Hence, our results argue that CAV1 promotes a rate-limiting step of DC migration to the LNs.

To define more precisely the underlying mechanism(s) by which CAV1 favored DC trafficking to LNs, different *in vitro* migration assays were performed. Our results show that neither amoeboid DC migration nor migration in confined environments were dependent on CAV1 expression (Figures 3A,B). However, results from the transwell assay (Figure 3C), which relies on migration through two-dimensional surfaces and passing through small pores, suggest that CAV1 promotes DC transmigration through afferent lymphatic vessels. First, DCs need to enter into lymphatic capillaries through preformed pores present in the basement membrane (41, 54), which are about 3 µm in diameter (55), but can be stretched to allow DC entry during transmigration (41, 44). Thus, in our experiments we used transwells with 8 µm size pores to maximize DC transmigration. After entering into the lumen of lymphatic capillaries, DCs need to migrate through the lymphatic endothelium surface following CCL21 gradients toward LNs (42). Hence, our results suggest that CAV1^−/−^ DCs are unable to appropriately enter and migrate through lymph vessels. Since these processes are dependent on cytoskeleton remodeling, impaired formation of actin-forming protrusions observed in CAV1^−/−^ DCs may provide an explanation for our results showing reduced *in vivo* and *in vitro* migration. Given that Rac1 modulates actin protrusion formation and DC migration (19, 45, 56), and that Rac1 activity is reduced in CAV1^−/−^ DCs, CAV1 may control DC migration by promoting Rac1 activity. It appears that formation of actin protrusions is a major consequence of Rac1 activity, which is linked to DC migration. As described by Benvenuti et al., DC migration *in vivo* was impaired in Rac1/2-deficient cells, as were rearrangements of actin cytoskeleton after blocking the Rac1 pathway (19). Moreover, CD81 promotes *in vitro* migration by increasing membrane protrusions *via* Rac1 activation (45). Also, CD37 ablation in DCs impairs cell migration *in vitro* (determined using a transwell assay), reduced dendrite formation *in vivo* and decreased Rac1 activity (56). Altogether, our results support the notion that CAV1 regulates DC migration by controlling actin cytoskeleton remodeling through activation of Rac1 and thus promotes efficient DC trafficking to LNs. However, further studies are required to precisely define the underlying mechanism(s).

The potential implications of CAV1 in promoting the ability of DCs to reach the LNs and initiate CD8^+^ T cell responses can be extrapolated to therapeutic interventions. DC-based immunotherapy has been proposed to have the potential to induce immune responses against cancer cells, but clinical trials have met with modest success (57, 58). Despite their initial poor clinical benefit, antigen-pulsed DC vaccines were recently shown to increase the breadth and diversity of melanoma neoantigen-specific T cells (59), and overall survival rates (57, 60) in stage IV melanoma patients, renewing the interest in employing DCs in clinical treatments. Migration to the LNs appears to represent a key limiting factor that determines the success of DC-based vaccination and the ability to induce T cell immune responses, which lead to favorable clinical outcomes (61). Indeed, DC migration to LNs seems to be a generally inefficient process in DC-based treatments (62), and as a consequence, the generation of specific adaptive immune responses is also suboptimal (63). Hence, the identification of molecular markers and the development of therapeutic strategies that improve DC trafficking need to be considered. Given our results showing that CAV1 promotes DC trafficking and thus the generation of more effective antitumor immune responses, CAV1 status may represent a novel marker for DC function and increasing CAV1 expression in DCs may help to improve DC-based immunotherapies.

In summary, our study demonstrates that CAV1 is upregulated in DCs upon maturation and promotes DC migration to LNs, probably by increasing actin cytoskeleton remodeling *via* Rac1 activation. While CAV1 expression in DCs is dispensable for CD8^+^ T cell activation *in vitro*, it enables DCs to reach the LNs to elicit effective antitumor CD8^+^ T cell responses *in vivo*. Therefore, our data identify a novel, hitherto unappreciated function of CAV1 in DCs with important consequences for basic aspects of DC biology that may open up a novel therapeutic window of opportunity to improve DC-based vaccines.

## Materials and Methods

### Mice

C57BL/6J CAV1 knockout mice (CAV1^−/−^, CAV1^tm1Mls^/J), C57BL/6-Tg(TcraTcrb)1100Mjb/J (OT-I), and C57BL/6J WT mice, mice were purchased from Jackson Laboratories (Bar Harbor, ME, USA). Mice were maintained at the SPF animal facility of Fundación Ciencia & Vida, where breeding and experimental procedures were carried out according to institutional guidelines. This study was carried out in accordance with the recommendations of the Comisión Nacional de Investigación Científica y Tecnológica, CONICYT.

### Bone Marrow (BM)- and Spleen-Derived DCs

Bone marrow-derived DCs were generated from flushed BM suspension from freshly dissected femurs and tibias. Cells were centrifuged for 5 min at 400 × *g*, treated with Red Blood Cells lysis buffer (BioLegend, San Diego, CA, USA) for 5 min, washed with PBS, centrifuged again and then cultured for 6 days in supplemented medium (RPMI medium; Hyclone, Logan, UT, USA) containing 10% fetal bovine serum (FBS, Hyclone, Logan, UT, USA), 20 ng/ml granulocyte-macrophage colony stimulating factor (BioLegend, San Diego, CA, USA), 1% non-essential amino acids, 1% l-glutamine, 1% penicillin–streptomycin, and 0.1% β-mercaptoethanol (Invitrogen, Carlsbad, CA, USA). Sp-DCs were purified from freshly isolated spleen using the EasySep Mouse CD11c Positive Selection Kit (StemCell, Vancouver, BC, Canada) according to the manufacturer’s instructions and then cultured in supplemented RPMI medium. Freshly isolated Sp-DCs or BM-DCs at day 6 were treated with LPS 100 ng/mL (*S. typhimurium*, L6143 Sigma-Aldrich, St. Louis, MO, USA) or TNF-α (20 ng/ml, BioLegend, San Diego, CA, USA) for different times as specified in each figure. In some experiments, BM-DCs were treated with LPS (100 ng/ml) together with anti-TNF-α blocking antibody (1 µg/ml, BioLegend, San Diego, CA, USA, clone MP6-XT22).

### Western Blotting

Dendritic cells (2 × 10^6^) were lysed in RIPA buffer (50 nM Tris–HCl, pH 7.4, 1% Triton-X, 0.5% Na-deoxycholate, 0.1% SDS, 150 mM NaCl, 2 mM EDTA, and 50 mM NaF) containing a protease inhibitor cocktail (cOmplete EDTA-free Protease Inhibitor Cocktail, Roche, Basel, Switzerland). Cell lysates were incubated for 15 min on ice and then centrifuged at 15,000 × *g* for 10 min at 4°C to obtain the supernatant. Protein concentrations were determined using the bicinchoninic acid protein assay kit (Thermo Scientific, Waltham, MA, USA) according to the manufacturer’s instructions. Samples’ concentration was normalized, and equal amount of samples were mixed with 6× SDS-polyacrylamide gel electrophoresis (SDS-PAGE) loading buffer [360 mM Tris–HCl (pH = 6,8), 60% glycerol, 12% SDS, 10% 2-mercaptoethanol, 0.03% Bromophenol Blue] heated to 95°C for 5 min and stored at −20°C until used. Then, 40 µg protein per sample was separated by SDS-PAGE (12% gel) at 120 V for 100 min in TGS buffer (25 mM Tris, 200 mM glycine, and 0.1% SDS) and then transferred to nitrocellulose membranes using a wet transfer Mini-Trans Blot system (Bio Rad, Hercules, CA, USA) at 400 mA for 90 min at 4°C in transference buffer (25 mM Tris, 200 mM glycine, 0.1% SDS, and 20% MeOH). The membranes were washed three times with PBS-Tween 0.05% and subsequently incubated with blocking solution (3% nonfat dry milk in PBS-Tween 0.05%) at room temperature for 1 h. Primary antibodies were incubated overnight at 4°C diluted in blocking solution: anti-CAV1 (1:4,000, BD Transduction Laboratories, Franklin Lakes, NJ, USA), anti-actin (1:5,000, Sigma-Aldrich, St. Louis, MO, USA), anti-GAPDH (1:5,000, Cell Signaling, Danvers, MA, USA), and anti-Rac1 (1:4,000, BD Transduction Laboratories, Franklin Lakes, NJ, USA). Then, the membranes were washed and incubated with anti-rabbit HRP-linked secondary antibody (1:2,000, Sigma-Aldrich, St. Louis, MO, USA) for 1 h at room temperature in blocking solution. Chemiluminescence was detected using the Supersignal West Pico kit (Thermo Scientific, Waltham, MA, USA) followed by exposure to photographic films (CL-XPosure Films, Thermo Scientific, Waltham, MA, USA). The digitized images were used for densitometry analysis using ImageJ software (NIH, Bethesda, MD, USA). CAV1 levels were normalized by actin or GAPDH levels, depending on the experiment.

### FITC Painting Assay

This procedure was performed as previously described (38). The back skin of mice was shaved and then inoculated on the right lateral flank with 12 µl of a 1% FITC solution (Isomer 1, Sigma-Aldrich, St. Louis, MO, USA) prepared in acetone (control). The left flank was inoculated with 12 µl of a 1% FITC solution prepared in acetone plus DBP (1:1) to induce skin irritation. After 20–22 h, mice were sacrificed, and draining inguinal LNs were obtained, and single cell suspensions were prepared using a solution containing collagenase IV (5 mg/ml, Gibco, Thermo Scientific, Waltham, MA, USA) and DNAse I (5 mg/ml, AppliChem, Maryland Heights, MO, USA) in RPMI supplemented with 0.5% FBS for 45 min at 37°C in a shaker bath. Then, the cells were stained with either anti-CD11c PE/Cy7-conjugated (clone N418, BioLegend, San Diego, CA, USA), anti-MHC-II APC/Cy7-congujated (clone M5/114.15.2, BioLegend, San Diego, CA, USA), or Zombie Aqua (BioLegend, San Diego, CA, USA) for 30 min and then fixed with a 2% paraformaldehyde solution and then evaluated by flow cytometry. The percentage of FITC^+^ cells in CD11c^+^MHC-II^high^ZA^neg^ was determined.

### DC Migration to Popliteal LNs

Bone marrow-derived DCs from WT or CAV1^−/−^ mice were stained with carboxyfluorescein succinimidyl ester (CFSE, BD Biosciences, Franklin Lakes, NJ, USA) or CTV (BD Biosciences, Franklin Lakes, NJ, USA), respectively, according to protocols provided by the manufacturer. Then, labeled WT and CAV1^−/−^ BM-DCs were mixed at a 1:1 ratio in PBS and 5 × 10^5^ total BM-DCs in 40 µl were injected into the footpad of the left lower extremity (footpad) of recipient WT mice. The exact percentage of WT and CAV1^−/−^ actually injected (input DC) in was determined by FACS as CTV^+^ or CFSE^+^ cells in the mix and used to calculate “migration index.” After 24 h postinjection, draining popliteal lymph nodes of each limb were obtained and digested as described earlier. Single cell suspensions were labeled with anti-CD11c conjugated with APC/Cy7 (clone N418, BioLegend, San Diego, CA, USA) and anti-MHC-II conjugated with PerCP (clone M5/114.15.2, BioLegend, San Diego, CA, USA) and then analyzed by flow cytometry. The number of cells that migrated (migration index) was calculated as follows:

$$\text{migration index}=\;\left\{\frac{\text{\% stained DC in PLN}/\text{\% stained DC in input}}{\text{\% WT DC in PLN\% WT DC in input}}\right\}.$$

The mean of the values for control condition was defined as 1 for further relativization.

### Transwell Assay

Transwell assays were performed in Boyden chambers (Transwell Costar, Thermo Scientific, Waltham, MA, USA, 6.5 mm diameter, 8 µM pore). The outer side of the membrane was coated with 2 µg/ml fibronectin for 18 h at 4°C. BM-DCs (2 × 10^4^ in 200 µl of RPMI medium containing 0.5% FBS) were seeded in the upper chamber, and the same medium containing CCL21 (20 ng/ml, BioLegend, San Diego, CA, USA) was added to the lower chamber to induce migration. After 1 h, the membranes were removed, washed, and stained with a solution containing 0.1% crystal violet in 2% ethanol and cells that migrated and adhered to the lower membrane surface were photographed under a microscope and counted. The migration index was calculated as follows:

$$\begin{array}{c} \text{migration index}=\text{ number of migrated DC}/\text{number of migrated WT DC in control}. \end{array}$$

The average of control condition was defined as 1 for further relativization.

### Migration in Microchannels

Bone marrow-derived DCs were prepared for migration in microchannels as previously described (64), and the experiments were conducted as published before (64). In brief, the cells were introduced into the fibronectin (10 µg/ml)-coated microchannels, without any mechanical or chemical stimulation. To assess the effect of LPS on DC migration in microchannels, BM-DCs were treated or not with LPS (1 µg/ml) for 30 min, followed by three rinses to wash out the LPS. After 5–6 h of LPS treatment, cells’ phase contrast images were recorded during 10–12 h at various positions in the chambers and with 2 min time lapses (to record multiple fields at low resolution for statistics) using an automated microscope (Nikon ECLIPSE TE1000-E and Olympus X71, with a Marzhauser motorized stage and an HQ2 Roper camera) equipped with an environmental chamber to control temperature, humidity, and CO_2_ (Life Imaging Services). The analysis of migration parameters was performed as described previously (64).

### Migration in Collagen Gels

Mature DCs were obtained by treating immature DCs with LPS (100 ng/ml) for 30 min and washing three times with supplemented medium. For collagen preparation, 120 µl of DCs (stock at 2 × 10^6^ million/ml) were carefully mixed with 205 µl of bovine type I collagen (stock 6 mg/ml) (Advanced BioMatrix, San Diego, CA, USA) and 13 µl of NaHCO_3_ (stock 7.5%) (Sigma-Aldrich, St. Louis, MO, USA). All solutions were previously equilibrated at 4°C. Then, the sample was loaded in a custom-made poly(dimethylsiloxane) (PDMS) “collagen chamber”. The chip was then incubated at 37°C for 30 min to allow collagen polymerization. To generate the CCL21 gradient, BM-DC medium containing 200 ng/ml of CCL21 (R&D Systems, Minneapolis, MN, USA) was added outside of the chamber. The cells were imaged by phase contrast at a frequency of 1 image every 2 min using a 10× objective. Images were processed to visualize cells by subtraction of the mean image of the whole movie at every time point, to obtain white objects on a dark background. Then cells were tracked as previously described (65).

### Confocal Microscopy and Image Acquisition

Bone marrow cells (2 × 10^5^) were cultured in 12-well plates containing 10 mm coverslips and differentiated into BM-DCs for 6 days using GM-CSF, as described. Cells were stimulated or not with LPS (100 ng/ml) for 24 h, washed three times with cold PBS and then fixed with 4% paraformaldehyde for 10 min at 4°C, washed again, permeabilized using Triton X-100 0.2% in PBS for 10 min, and then blocked with a PBS-BSA 3% solution for 1 h at room temperature. The cells were incubated with anti-CAV1 antibody (1: 200) overnight at 4°C, washed three times with PBS and then incubated 2 h at room temperature in the dark with anti-rabbit second antibody conjugated with Alexa Fluor 488 (Thermo Scientific, Waltham, MA, USA) in a 1: 500 dilution together with 500 nM phalloidin rhodamine (Sigma-Aldrich, St. Louis, MO, USA) and 100 nM 4′,6-diamidino-2-phenylindole (Sigma-Aldrich, St. Louis, MO, USA). Coverslips were washed and mounted on microscope slides with Mowiol 4-88 (Sigma-Aldrich, St. Louis, MO, USA), and samples were visualized with an Olympus IX81 DSU microscope and analyzed with ImageJ software. Membrane protrusions were counted manually for at least 10 cells per condition in two experiments.

### Rac1-GTP Pull-down Assay

Rac1-GTP pull-down assays were performed as described previously (27). Briefly, cells were lysed in a buffer containing 25 mM HEPES (pH 7.4), 100 mM NaCl, 5 mM MgCl_2_, 1% NP-40, 10% glycerol, 1 mM dithiothreitol, and protease inhibitors. Extracts were incubated for 5 min on ice and clarified by centrifugation (10,000 × *g*, 1 min, 4°C). Supernatants were used for pull-down assays with 50 µg of GST-PAK1 pre-coated GSH beads per condition. Beads were incubated with supernatant for 15 min at 4°C in a rotating shaker. Thereafter, beads were collected, washed with lysis buffer containing 0.01% NP-40. Samples were separated by SDS-PAGE (12% acrylamide) and analyzed by Western blotting using anti-Rac1 (1:1,000) antibody from Transduction Laboratories (Lexington, KY, USA).

### T Cell Proliferation Assay

Wild-type or CAV1^−/−^ DCs were pulsed with different amounts of OVA_257–264_ peptide (10, 1, or 0.1 µg/ml) for 4 h and then washed. 5 × 10^3^ DCs were cocultured with 1 × 10^5^ CFSE-labeled (as described before) CD8^+^ T cells purified from the spleen of OT-I mice using the EasySep CD8^+^ T Cell Enrichment Kit (StemCell, Vancouver, BC, Canada). Proliferation of CD8^+^ T cells was evaluated by assessing CFSE dilution by flow cytometry in the CD3^+^CD8^+^Vα2^+^ population using PerCP-conjugated anti-CD3 (clone 145-1211, BioLegend, San Diego, CA, USA), APC-conjugated anti-CD8 (clone 53-6.7, BioLegend, San Diego, CA, USA), and Pacific Blue-conjugated anti-Vα2 (clone B20.1, BioLegend, San Diego, CA, USA).

### DC Staining and Cytokine Secretion

Wild-type or CAV1^−/−^ DCs were stimulated or not with LPS (100 ng/ml) for 24 h and then stained for 30 min at 4°C in 50 μl of PBS-BSA 2% solution with the following antibodies (all from BioLegend, San Diego, CA, USA): anti-CD11c APC-conjugated (clone N418, dilution 1:250), anti-CD40 PE-conjugated (clone 3/23, dilution 1:125), anti-CD80 FITC-conjugated (clone 16-10A1, dilution 1:250), anti-CD86 PE/Cy7-conjugated (clone GL-1, dilution 1:250), anti-CD38 Alexa Fluor 488-conjugated (clone 90, dilution 1:200), anti-PD-L1 PE/Cy7-conjugated (clone 10F.9G2, dilution 1:250), anti CD14 PE-conjugated (clone Sa14-2, dilution 1:100), anti-CCR7 Brilliant Violet 421-conjugated (clone 4B12, dilution 1:100), anti-MHC-I PE-conjugated (clone M1/42, dilution 1:125), and anti-MHC-II PerCP-conjugated (clone M5/114.15.2, dilution 1:250). Zombie Aqua (BioLegend) was used to determine cell viability (1:500 dilution). Non-specific binding was blocked by mouse Fc receptor blocking (BioLegend, clone 93, dilution 1:100). Then, the cells were washed and fixed with 2% paraformaldehyde PBS and analyzed by flow cytometry. Approximately 10,000 events in the MHC-II^+^CD11^+^ gate were recorded per sample. Samples were acquired in a FACSCanto II cytometer (BD Bioscience), and the data analyzed using FlowJo version X (Tree Star Inc.). If correspond, the average of control condition was defined as 1 for further relativization. For the ELISA experiments, the supernatants of stimulated DCs were used to determine secretion of IL-6 (capture antibody: clone MP5-20F3; detection antibody: clone MP5-32C11); IL-12 (capture antibody: clone C18.2; detection antibody: clone C17.8), and TNF-α (capture antibody: clone MP6-XT22; detection antibody: C19.2), following the manufacturer’s instructions (BioLegend, San Diego, CA, USA).

### DC Immunization and Evaluation of CD8^+^ T Cell Responses

Wild-type or CAV1^−/−^ DCs pulsed with OVA_257–264_ peptide (10 µg/ml for 4 h) were washed three times with cold PBS and then injected intravenous (i.v., 10^6^ cells) into recipient mice. PBS was used as vehicle control. Seven days after immunization, blood samples were obtained, and red blood cells were removed by using Red Blood Cells lysis buffer as described before. The samples were split in two fractions: one used for intracellular staining and the second for multimer staining. For intracellular cytokine staining, the cells were washed with PBS and stimulated *ex vivo* with OVA_257–264_ peptide (2.5 µg/ml) in supplemented RPMI. After 2 h, Golgi plug (brefeldin A) was added (1 µl/ml, BD Biosciences, Franklin Lakes, NJ, USA) for the last 6 h. Cells were first incubated with labeled with Fc receptor blocking (BioLegend, clone 93) and then labeled with the following antibodies (all from BioLegend, San Diego, CA, USA): PerCP-conjugated anti-CD3 (clone 145-1211), APC/Cy7-conjugated anti-CD8 (clone 53-6.7), plus Zombie aqua (as viability dye). Then cells were fixed and permeabilized using BD Cytofix/Cytoperm Kit (BD Biosciences, Franklin Lakes, NJ, USA) according to the manufacturer’s instructions. Intracellular staining was performed using PE-conjugated anti-INF-γ (clone XMG1.2) and APC-conjugated anti-TNF-α (clone MP6-XT22) antibodies. For multimer staining, cells were incubated with Fc receptor blocking and labeled PerCP-conjugated anti-CD3 (clone 145-1211), APC/Cy7-conjugated anti-CD8 (clone 53-6.7), Zombie aqua (as described earlier), and APC-conjugated MHC class I H-2Kb dextramer loaded with OVA_257–264_ peptide (SIINFEKL), according to the manufacturer’s instructions (Immudex, Copenhagen, Denmark).

### Tumor Challenge

After 12 days from DC immunization, mice were injected subcutaneously with B16F10-OVA tumor cells (2.5 × 10^5^ cells in PBS, 95% > viability, 60–80% confluence at harvesting day). The evaluation of tumor size (length, width, and height) started 5 days after challenge, and the volume was calculated as (length × width × height)/2. Mice were sacrificed if one of the measures exceeded 15 mm, to avoid unnecessary suffering. The tumor size was plotted against time post challenge, and the animal survival was plotted as Kaplan–Meier survival curve.

### Statistical Analysis

Experimental data are presented as the mean ± SD or mean ± SEM of the number of experiments indicated as “*n*” or as representative results of at least two independent experiments. For determination of significance, data sets of two conditions were analyzed using Student’s *t*-test and Turkey’s *post hoc* analysis; for multiple data sets, one-way analysis of variance was used (ANOVA) and Bonferroni’s *post hoc* applied. Tumor growth was compared using the Wilcoxon rank sum test, and survival curves (Kaplan–Meier survival curve showing tumor-free survival) were compared using the Mantel–Cox test. A *p* value <0.05 was considered a statistically significant difference between the data compared (***p ≤ 0.001; **p ≤ 0.01, and *p ≤ 0.05).

## Ethics Statement

This study was carried out in accordance with the recommendations of the “Guidelines for the welfare and use of animals in cancer research, Committee of the National Cancer Research Institute”. The protocol was approved by the “Committee of Bioethics and Biosafety” from Fundación Ciencia & Vida.

## Author Contributions

AL, CO, and AQ designed research; CO, SC-G, FG-C, PV, HM, ND-V, and JD performed research; CO, SC-G, FG-C, and PV prepared figures: CO, SC-G, FG-C, PV, AQ, and AL analyzed and interpreted the data; AL-D, RP, and FS-O contributed tools and discussed results; and CO, AQ, and AL wrote the paper.

## Conflict of Interest Statement

The authors declare that the research was conducted in the absence of any commercial or financial relationships that could be construed as a potential conflict of interest.
