# Supplementary material for: Caveolin-1 Expression Increases upon Maturation in Dendritic Cells and Promotes Their Migration to Lymph Nodes Thereby Favoring the Induction of CD8+ T Cell Responses
Source: Front Immunol. 2017 Dec 13;8:1794. doi: 10.3389/fimmu.2017.01794 (PMC5733362; doi:10.3389/fimmu.2017.01794)
Supplement: Supplementary file 1 [file Image_1.pdf]

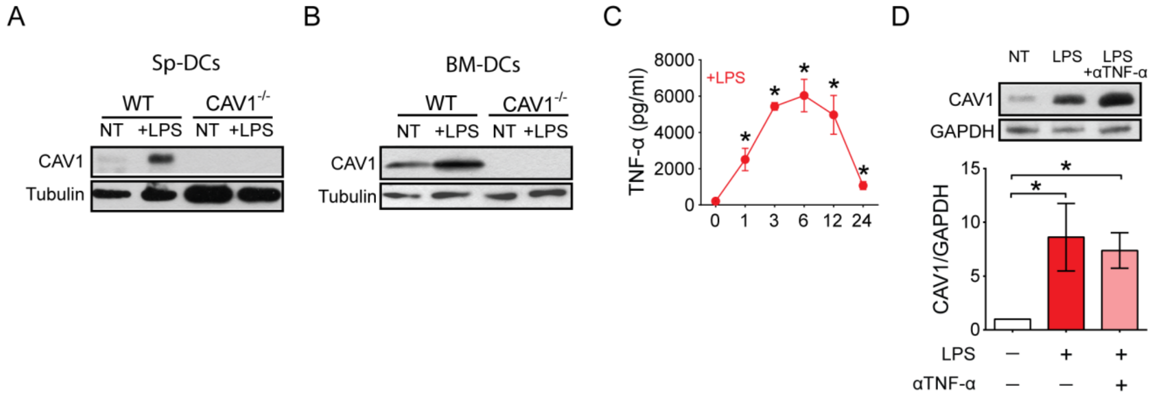

S1. Specific detection of caveolin-1 expression in DCs. Sp-DCs (**A**) and BM-DCs (**B**) were treated with LPS (100 ng/ml) for 24 h or 6 h, respectively, and caveolin-1 expression was determined by Western blotting. Bars are the mean ± SEM (* = p<0.05 v/s non-treated condition (NT) or as indicated, n = 3). (**C**) Time-dependent TNF-α secretion induced by LPS**.** BM-DCs were stimulated with LPS (100 ng/ml) 0, 1, 3, 6, 12 or 24 h and then supernatants were collected and TNF-α secretion was determined by ELISA. Dots are the mean ± SEM (* = p<0.05 v/s 0h treatment, n = 3). (**D**) TNF-α blockade following LPS- stimulation. BM-DCs were treated with LPS or a combination of TNF-α blocking antibody (5 µg/ml) plus LPS for 6 h and caveolin-1 expression was determined by Western blotting. Bars or dots are the mean ± SEM (* = p<0.05 v/s NT or as indicated, n = 3).
