# Supplementary material for: Caveolin-1 Expression Increases upon Maturation in Dendritic Cells and Promotes Their Migration to Lymph Nodes Thereby Favoring the Induction of CD8+ T Cell Responses
Source: Front Immunol. 2017 Dec 13;8:1794. doi: 10.3389/fimmu.2017.01794 (PMC5733362; doi:10.3389/fimmu.2017.01794)
Supplement: Supplementary file 2 [file Image_2.pdf]

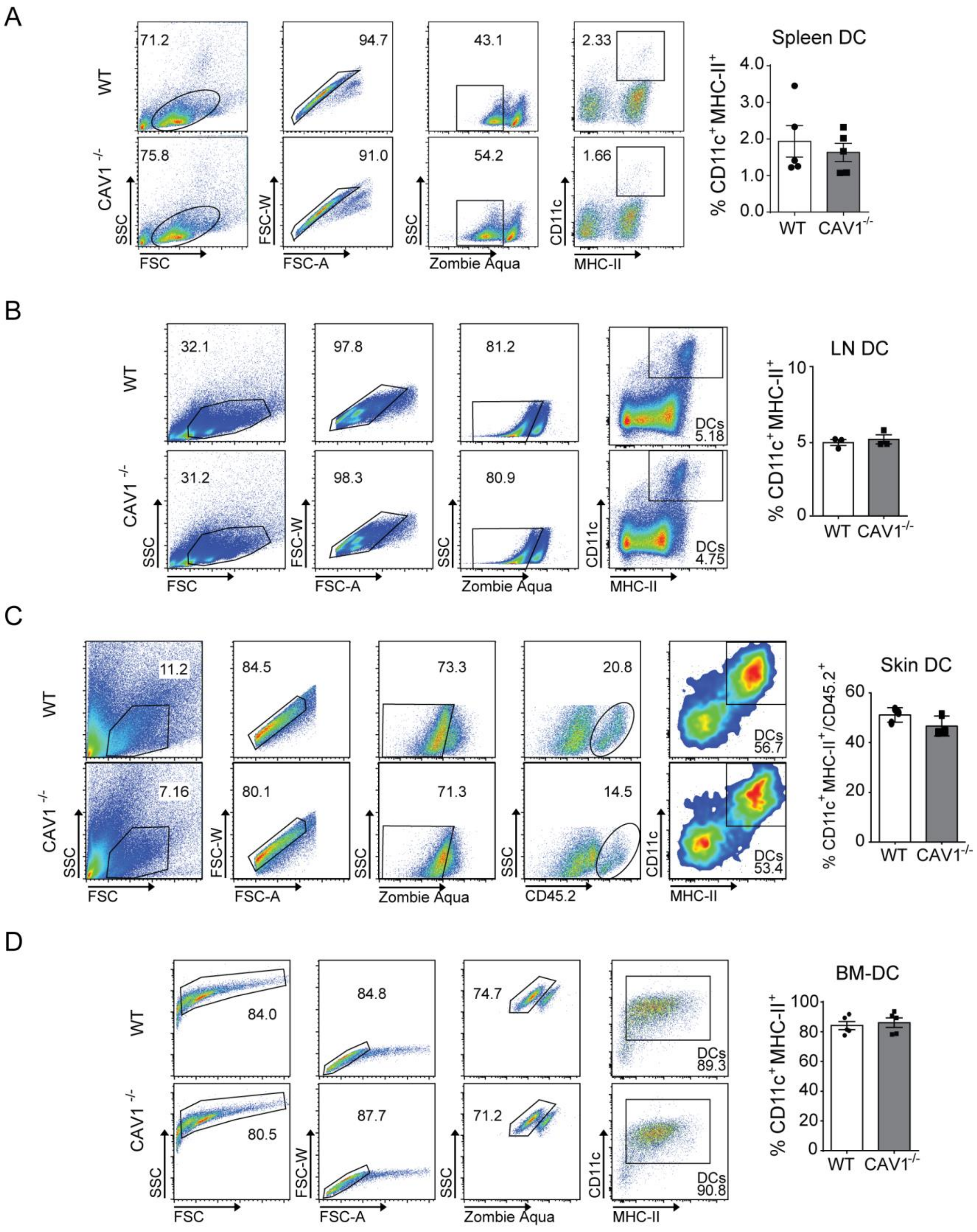

S2. Caveolin-1 is not involved in DC differentiation. (**A**),(**B**) DC percentage in spleen (**A**) or lymph nodes (**B**). WT and CAV1$^{-/-}$ mice were sacrificed and spleen and inguinal lymph nodes were obtained and digested in RPMI plus collagenase IV (5 mg/ml) and DNAse I (5 mg/ml) for 45 min. Then, cells were stained (30 min) with anti-CD11c, anti-MHC-II and Zombie Aqua, washed and fixed with a PBS/paraformaldehyde 4% solution and then evaluated by flow cytometry. The percentage of cells CD11c^{+}MHC-II^{high}ZA^{neg} was assessed. Each dot represents one animal and the bar is the mean (n = 5 for spleen, n=3 for lymph node). (**C**) Skin from WT and CAV1$^{-/-}$ mice skin was processed to obtain a single cell

suspension. Briefly, WT and CAV1$^{-/-}$ mice were sacrificed, shaved and skin pieces of 1 cm^2 were digested using a solution containing collagenase IV (5 mg/ml) and DNAse I (5 mg/ml) for 1 h. Then, cells were stained (30 min) with anti-CD45, anti-CD11c, anti-MHC-II and Zombie Aqua, washed and fixed with a PBS/paraformaldehyde 4% solution and then evaluated by flow cytometry. The percentage of cells $CD45^+CD11c^+MHC\text{-}II^{high}ZA^{neg}$ was assessed. Each dot represents one animal and the bar is the mean (n = 3). (**D)** Bone marrow cells from WT o CAV1$^{-/-}$ mice were cultured for 6 days in medium containing GM-CSF (20 ng/ml) to generate BM-DCs. Then, cell viability (Zombie Aqua) and $CD11c^+MHC\text{-}II^+$ was assessed by flow cytometry. No differences were observed in CD11c expression between BM-DCs from both mouse strains (n=5).
