# Supplementary material for: Caveolin-1 Expression Increases upon Maturation in Dendritic Cells and Promotes Their Migration to Lymph Nodes Thereby Favoring the Induction of CD8+ T Cell Responses
Source: Front Immunol. 2017 Dec 13;8:1794. doi: 10.3389/fimmu.2017.01794 (PMC5733362; doi:10.3389/fimmu.2017.01794)
Supplement: Supplementary file 3 [file Image_3.pdf]

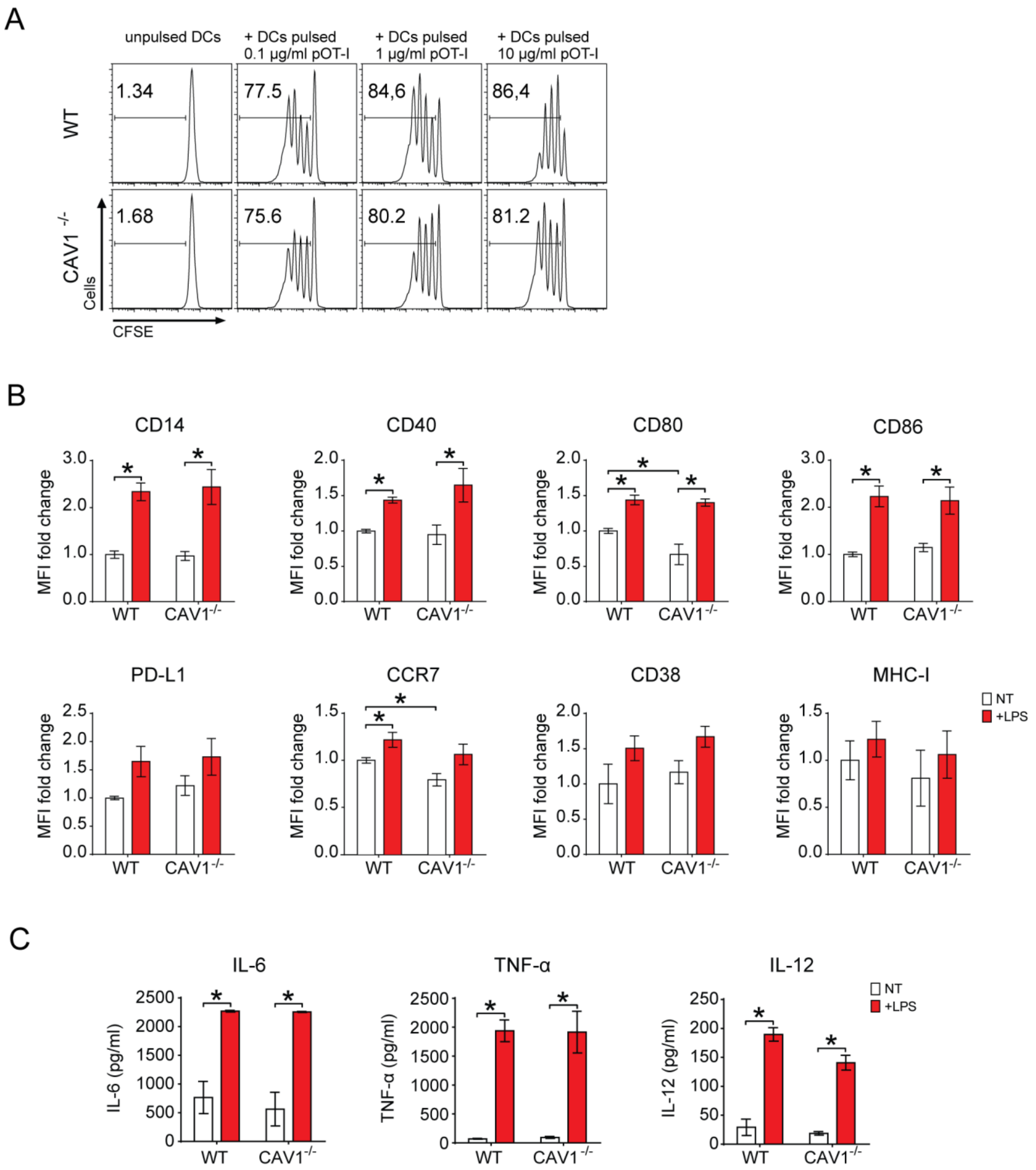

S3. Caveolin-1 is not involved in DC maturation and antigen-specific activation of CD8+ T cells *in vitro*. (**A**) WT or CAV1$^{-/-}$ DCs were loaded with OVA$_{257\text{-}265}$ peptide and co-cultured with OT-I CD8+ T cells (1:20, DC:T cell). *Ex vivo* OT-I CD8+ T cell proliferation induced by OVA$_{257\text{-}265}$ peptide-pulsed DCs was evaluated after 3 days of co-culture. Representative histograms showing proliferation profiles of OT-I CFSE^{+}CD3+CD8+Vα2^{+} populations for each group are displayed (n=3). (**B**) Expression of co-stimulatory markers in WT or CAV1$^{-/-}$ DCs. The expression of C14, CD40, CD80, CD86, PD-L1, CCR7, CD38 and MHC-I was determined by flow cytometry. Data are the mean of relative expression ± SD compared with non-treated (NT) (* = $p<0.05$, n = 3-6).

(**C**) Cytokine secretion (IL-6, IL-12 and TNF-α) by DCs after stimulation for 24 h with LPS (100 ng/ml) was determined by ELISA. Bars are the mean ± SD compared with non-treated (NT) (* = $p<0.05$, n=3).
